# Population Genetics of *Seriola lalandi* Confirm Hemispheric Divergence and Reveal Genetic Structure in the Temperate Northern Pacific and Between Wild and Captive‐Reared Fish

**DOI:** 10.1002/ece3.74200

**Published:** 2026-08-20

**Authors:** Eduardo Martínez‐Matus, Felipe Aguilera, Raquel Muñiz‐Salazar, Fabiola A. Sepúlveda, M. Teresa González, Cristian Araneda‐Tolosa, Claudia Farfán, Fabiola Lafarga‐De la Cruz

**Affiliations:** ^1^ División de Oceanología, Departamento de Acuicultura Centro de Investigación Científica y de Educación Superior de Ensenada (CICESE) Ensenada Baja California México; ^2^ Instituto de Biología, Facultad de Ciencias Pontificia Universidad Católica de Valparaíso Valparaíso Chile; ^3^ Departamento de Bioquímica y Biología Molecular, Facultad de Ciencias Biológicas Universidad de Concepción, Barrio Universitario s/n Concepción Chile; ^4^ Laboratorio de Epidemiología y Ecología Molecular, Escuela Ciencias de la Salud Universidad Autónoma de Baja California Ensenada Baja California México; ^5^ Instituto de Ciencias Naturales Alexander von Humboldt, Facultad de Ciencias del Mar y Recursos Biológicos Universidad de Antofagasta Antofagasta Chile; ^6^ Departamento de Producción Animal, Facultad de Ciencias Agronómicas Universidad de Chile La Pintana Santiago Chile

**Keywords:** aquaculture, broodstock management, genetic conservation, genetic diversity, genetic structure, yellowtail kingfish

## Abstract

The Yellowtail Kingfish ( *Seriola lalandi* ) is a widely distributed marine species of high commercial value for both fisheries and aquaculture. Although the taxonomic status of *S. lalandi* remains unresolved, this study examined its genetic structure across two Pacific Ocean regions and evaluated the genetic differentiation between wild and captive‐reared fish. Globally, our results demonstrated two genetically differentiated groups of *S. lalandi* : one restricted to the Temperate Northern Pacific (Mexico‐United States) and another to Temperate South America (Chile), with pronounced divergence (*F*_ST_ = 0.208). Among wild populations, individuals from the Northern Hemisphere displayed greater heterozygosity compared to those from the Southern Hemisphere (Chile). We also detected fine‐scale population substructure within the Northern Pacific (*F*_ST_ = 0.078), along with consistent genetic differentiation between wild fish and captive‐reared stocks in both Chile (*F*_ST_ = 0.049) and Mexico (*F*_ST_ = 0.120). Together, these findings support the hypothesis of two independent evolutionary lineages in *S. lalandi* and challenge the current notion that a single northern stock (referred to as *Seriola dorsalis* ) exists in the Temperate Northern Pacific, highlighting the need for region‐specific management strategies for fisheries and aquaculture.

## Graphical abstract

We investigated the genetic diversity and population structure of *Seriola lalandi* across the Pacific Ocean, including wild and captive‐reared individuals. Our results revealed pronounced hemispheric genetic differentiation and previously undetected substructure within the Temperate Northern Pacific, along with consistent differences between wild and hatchery‐reared fish. These findings support the presence of distinct evolutionary lineages and highlight the need for region‐specific management and breeding strategies.

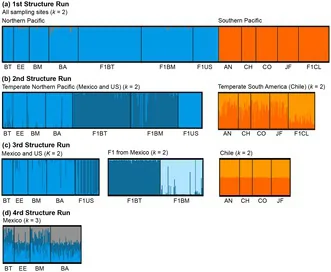

## 1 Introduction

The genus *Seriola* comprises marine teleost fishes of the family Carangidae, commonly known as amberjacks, which are distributed worldwide and inhabit both deep offshore waters, such as those adjacent to oceanic islands, and shallower coastal areas (Nakada 2008; Ben‐Aderet et al. 2020). This genus includes several species that are highly valued in recreational and commercial fisheries and in aquaculture operations (Ben‐Aderet et al. 2020; Rotman et al. 2021). The Yellowtail Kingfish ( *Seriola lalandi* Valenciennes, 1833) is particularly valued due to its fast growth rate, efficient feed conversion rate, high market demand, and suitability for offshore cage and land‐based farming operations (Symonds et al. 2014; Shu‐Chien et al. 2025). Currently, *S. lalandi* aquaculture production is led by Japan, where the industry depends on juveniles caught in the wild, and by Australia, where it depends solely on captive‐reared fish, with the grow‐out phase in both countries mainly conducted in sea cages (Nakada 2008; Sicuro and Luzzana 2016; Rotman et al. 2021; Clean Seas Seafood Limited 2024).

Currently, the population structure of *S. lalandi* is a matter of debate at both regional and global scales (Mechaly et al. 2025). Studies based on mitochondrial, nuclear, microsatellite, and SNP markers have found significant divergence among populations from the Northern and Southern Hemispheres (Nugroho et al. 2001; Martinez‐Takeshita et al. 2015; Purcell et al. 2015; Premachandra et al. 2017; Ai et al. 2021; Cui et al. 2023), as well as notable morphometric differences among geographically distinct *S. lalandi* populations (Martinez‐Takeshita et al. 2015; Ai et al. 2021). A decade ago, Martinez‐Takeshita et al. (2015) addressed the question of whether some *S. lalandi* populations might actually consist of distinct species. However, after analyzing *S. lalandi* populations from the western Pacific (Japan and Australia) and eastern Pacific (United States, Mexico, and Chile), Premachandra et al. (2017) did not find genetic differences among regions that were sufficient to support subdivision of *S. lalandi* into several species.

In the Southern Hemisphere, the results of regional‐scale genetic analyses have suggested that *S. lalandi* can be separated into two groups: a southern Pacific (i.e., Chile and Australia) population and a southern Atlantic (i.e., South Africa) population (Purcell et al. 2015; Swart et al. 2016). Moreover, the population structure of *S. lalandi* has been the subject of multiple studies along the coasts of Australia and New Zealand (Miller et al. 2011; Premachandra et al. 2017), South Africa (Swart et al. 2016), Chile (Fernández et al. 2015; Sepúlveda and González 2017), and the United States (Baxter 1960; Purcell et al. 2015). Notably, population structure has been detected in *S. lalandi* from Australia and New Zealand, which was mainly attributable to geographical or environmental barriers (Miller et al. 2011). In addition, high gene flow and a single panmictic population of *S. lalandi* have been found in South Africa and Chile (Fernández et al. 2015; Swart et al. 2016), even though Sepúlveda and González (2017) demonstrated genetic differentiation among years and sampling locations. However, in the Temperate Northern Pacific, specifically in Mexico and the United States, the population structure of *S. lalandi* remains poorly understood and inadequate for delineating fish stocks (Baxter 1960; Purcell et al. 2015; Ben‐Aderet et al. 2020).

On the Pacific coast of North and South America, *S. lalandi* aquaculture is currently under development, albeit with great expectations fueled by a worldwide growing demand. To date, aquaculture efforts, which are primarily aimed at producing hatchery‐reared fish, have been centered in three countries: the United States, Mexico, and Chile (Rotman et al. 2021; Mechaly et al. 2025). However, the potential increase in the translocation of stocks among national and international hatcheries, a common practice in aquaculture, is worrisome due to the controversies surrounding proper species identification and the population genetics of the putative *S. lalandi* populations in these locations (Miller et al. 2011; Purcell et al. 2015). Indeed, this movement of genetic material may inadvertently impact local wild populations, leading to inbreeding or outbreeding depression (Maheshwari and Barbash 2011; Rollinson et al. 2014; Purcell et al. 2015). Despite observed unequal reproduction in cultured *S. lalandi* populations (Dettleff et al. 2020; Schmidt et al. 2021), comparative genetic studies for wild and cultivated *S. lalandi* are limited. For example, Cui et al. (2023) reported no evidence of genetic differences between *S. lalandi* from wild and cultured populations in China; conversely, they reported clear genetic divergence between wild fish and second‐generation hatchery progeny produced from captive‐born broodstock (F2) in Australia. However, given the apparent global distribution and commercial movements of *S. lalandi* , especially from the Southern to Northern Hemisphere (Miller et al. 2011; Premachandra et al. 2017; Dettleff et al. 2020), an accurate understanding of the genetic relatedness between wild populations and captive‐reared stocks is crucial.

In the present study, we hypothesized that *S. lalandi* would exhibit a hierarchical genetic structure across the Pacific Ocean, characterized by marked genetic divergence between the Northern and Southern Hemispheres and finer‐scale population structure within the Temperate Northern Pacific. We further expected first‐generation hatchery‐reared offspring produced from wild broodstock (F1) to exhibit detectable genetic differences from wild populations, reflecting unequal parental contribution during broodstock reproduction. Accordingly, we aimed to evaluate the genetic diversity and population structure of *S. lalandi* based on 497 wild and captive‐reared individuals and to assess the genetic similarity of several F1 groups to wild *S. lalandi* , which is necessary for determining the potential impacts of unintended aquaculture escapees. To achieve these aims, we evaluated global and regional patterns of genetic structure in *S. lalandi* to assess the level of relatedness and migration among fish from two marine realms of the Pacific Ocean: Temperate South America (Chile) and the Temperate Northern Pacific (Mexico‐United States). Next, we analyzed the genetic similarity between wild and captive‐reared *S. lalandi* from the coasts of Mexico and Chile. Using both Bayesian and non‐Bayesian approaches, we identified distinct genetic stocks in the Pacific Ocean, confirming genetic differentiation between *S. lalandi* populations in the Northern and Southern Hemispheres. At the regional scale, we found at least two subpopulations in the Temperate Northern Pacific and genetic differences between wild and captive‐reared fish, which highlights the need to improve the management of *S. lalandi* breeding and aquaculture programs.

## 2 Materials and Methods

### 2.1 Animal Ethics Statement

According to the Red List of Threatened Species of the International Union for Conservation of Nature, *S. lalandi* is considered a species of Least Concern (Smith‐Vaniz and Williams 2015). All animal procedures were conducted in accordance with the *Ley Federal de Sanidad Animal* (SADER 2024; Mexico) and European Council Directive 2010/63/EU (2010), which regulates the care and use of animals in scientific research. Fin clips from wild *S. lalandi* were collected non‐lethally, and all fish were released immediately after sampling. To minimize stress and suffering, hatchery‐reared juveniles were euthanized by anesthetic overdose (MS‐222; tricaine methanesulfonate) in accordance with the protocols of the American Veterinary Medical Association (AVMA 2020) and Ocean Baja Labs (Erendira, Baja California, Mexico). This study did not involve the use of animals at *Centro de Investigación Científica y de Educación Superior de Ensenada* (CICESE), Baja California, Mexico.

### 2.2 Study Sites, Sample Collection, and DNA Extraction

For clarity, in this study, F1 refers to first‐generation hatchery‐reared fish produced from wild broodstock maintained under captive breeding conditions, and F2 refers to second‐generation hatchery progeny produced from captive‐born broodstock.

**FIGURE 1:**
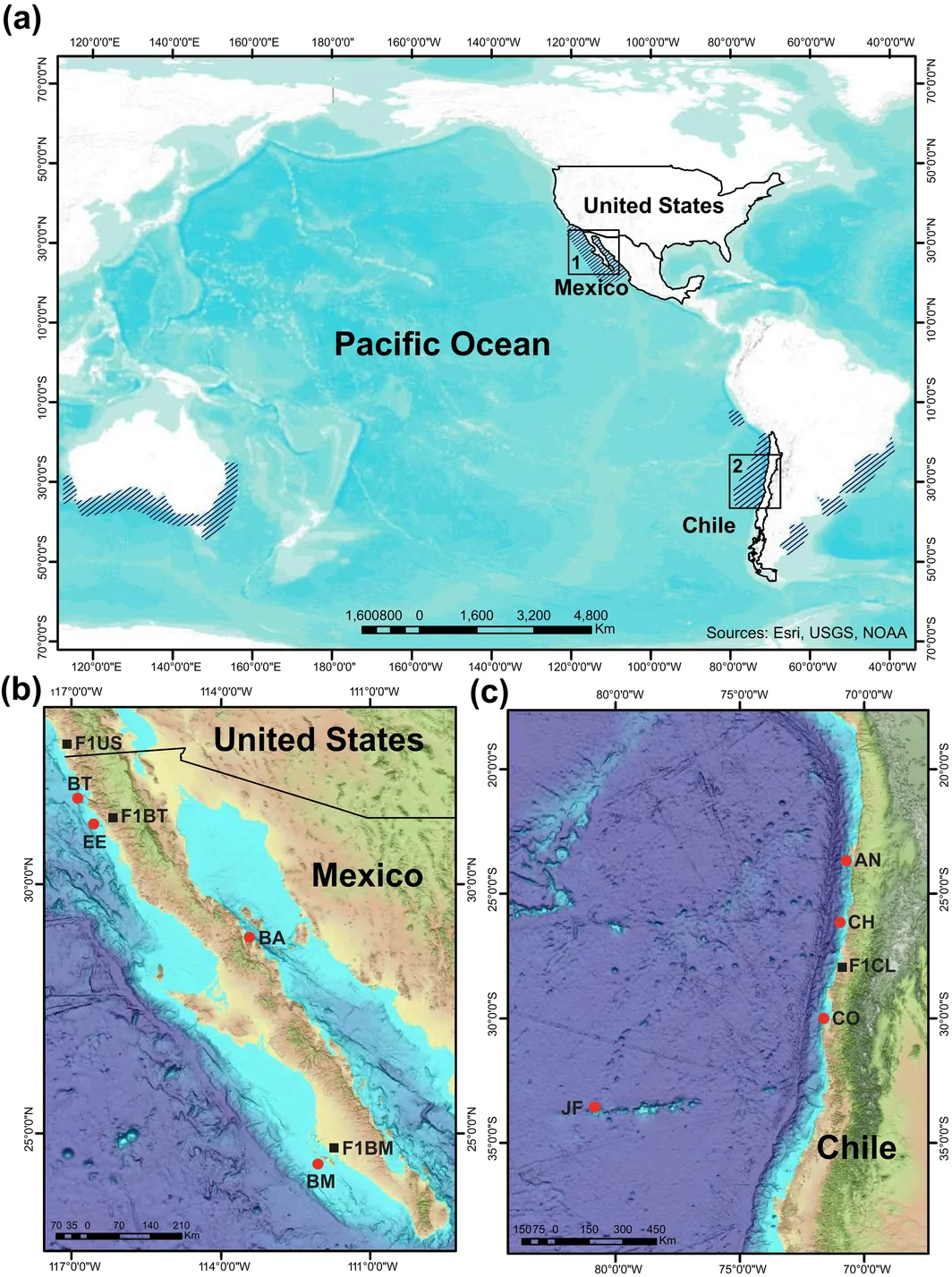
Sampling locations of wild and captive‐reared *Seriola lalandi* included in this study. Lines indicate the reported geographic distribution of *S. lalandi* *sensu lato*. (a) Regional distribution of sampling sites in the Pacific Ocean, including Temperate Northern Pacific (Mexico‐United States) and Temperate South America (Chile). (b) Locations in the Temperate Northern Pacific are shown with red and black markers. (c) Locations in Temperate South America are shown with red and black markers. Wild *S. lalandi* locations (red markers): Bahia Todos Santos (BT), Ejido Erendira (EE), Bahia Magdalena (BM), Bahia de los Angeles (BA), Antofagasta (AN), Chañaral (CH), Coquimbo (CO), and Juan Fernandez (JF). Captive‐reared *S. lalandi* stock (black markers): Bahia Todos Santos (F1BT), Bahia Magdalena (F1BM), United States (F1US), and Chile (F1CL). Maps generated using ArcGIS v. 10.2 with coastline data downloaded from Natural Earth (www.naturalearthdata.com).

Wild *S. lalandi* were obtained from 4 locations in Mexico (Bahia Todos Santos [BT], Ejido Erendira [EE], Bahia Magdalena [BM], Bahia de Los Angeles [BA]) and 4 locations in Chile (Antofagasta [AN], Chañaral [CH], Coquimbo [CO], and Juan Fernandez [JF]). Caudal fin clips were obtained from 336 *S. lalandi* adult specimens collected from these wild populations. In addition, 313 captive‐reared F1 *S. lalandi* juveniles were obtained from hatcheries located in Mexico (Bahia Todos Santos [F1BT] and Bahia Magdalena [F1BM]), the United States (F1US), and Chile (F1CL) (Figure 1 and Table S1). Our previously published partial dataset (Premachandra et al. 2017), comprising 76 individuals from Mexico and 50 from Chile, was reanalyzed in this study. We further expanded the microsatellite dataset by genotyping an additional 263 individuals from Mexico, 220 from Chile, and 40 from the United States (Table S1). All samples were preserved in 96% ethanol and stored at −20°C. Total DNA was extracted from tissue samples using the DNeasy Blood and Tissue kit (QIAGEN, Valencia, USA) following the instructions of the manufacturer, eluted into 50 μL of nuclease‐free water, and stored at −20°C at CICESE for subsequent analysis.

### 2.3 PCR Amplification and Microsatellite Genotyping

Six species‐specific microsatellite loci developed for *S. lalandi* (*Sel001*, *Sel002*, *Sel008*, *Sel011*, *Sel017*, and *Sel019*) (Whatmore et al. 2013; Premachandra et al. 2017) and three microsatellite markers isolated from *S. dumerili* (*Sdu21*, *Sdu32*, and *Sdu46*) (Renshaw et al. 2006, 2007) were used in this study. PCR amplifications and microsatellite fragment analysis were performed as previously reported (Whatmore et al. 2013), and genotypes were scored using GeneMarker (SoftGenetics, State College, USA). To minimize genotyping errors, samples that did not amplify or that yielded low‐quality results were reanalyzed, which allowed us to identify the high‐quality peaks necessary for scoring valid alleles. All PCR amplifications and microsatellite genotyping were performed at the Genecology Research Centre of the University of the Sunshine Coast, Australia.

### 2.4 Microsatellite Suitability

Microsatellite genotyping results were processed using GenAlEx v. 6.503 (Peakall and Smouse 2012) to produce input data for subsequent analyses. Possible allele assignment errors were checked using MICRO‐CHECKER v. 2.2.3 (van Oosterhout et al. 2004), and the null allele frequency (Table S2) was estimated through the maximum likelihood method implemented in ML‐Null software (Kalinowski and Taper 2006). ML‐RELATE (Kalinowski et al. 2006) was used to estimate relatedness among individuals from each location; in this study, wild individuals with relatedness values greater than 0.45 were excluded.

To detect outlier loci, we used BayeScan v. 2.1 (Foll and Gaggiotti 2008), which implements a Bayesian method to estimate locus‐specific *F*_ST_ values (alpha). Analyses were run with a burn‐in of 50,000 iterations, a thinning interval of 10, a posterior sample size of 1000,000, and 20 pilot runs of 5000 iterations each. To improve the accuracy of loci detection while accounting for hierarchical population structure, samples were grouped a priori into two major biogeographic units (i.e., Northern Hemisphere and Southern Hemisphere) (Excoffier et al. 2009). This grouping was based on the geographic distribution of the sampled populations and previous evidence of hemispheric genetic divergence in *S. lalandi* (Martinez‐Takeshita et al. 2015). Loci under potential selection were identified using a false discovery rate (FDR) threshold of 1%.

### 2.5 Genetic Diversity Analysis

Genetic diversity estimates of alleles per locus (*N*_A_), expected heterozygosity (*H*_E_), observed heterozygosity (*H*_O_), and Hardy–Weinberg equilibrium (HWE) were calculated using the default Markov chain parameters implemented in GenAlex v. 6.503 (Peakall and Smouse 2012). Deviations from HWE were tested for each wild population and captive‐reared stock based on differences between *H*_E_ under random mating and *H*_O_ in the studied populations. The statistical significance of HWE departures was assessed using Chi‐squared tests implemented in GenAlex v. 6.503 (Peakall and Smouse 2012) with a significance level of *α* = 0.05 (*p* < 0.05). Allelic richness (*A*_R_) and private allelic richness (*A*_P_) for each location were calculated with HP‐RARE (Kalinowski 2005), which employs a rarefaction approach to account for differences in sample size. Inbreeding coefficients (*F*_IS_) per locus were assessed using the Weir and Cockerham estimator (Weir and ockerham 1984), implemented in FSTAT v. 2.9.4 (Goudet 1995) with 1000 sequential randomizations. Bonferroni correction was applied to adjust for multiple comparisons (Rice 1989). The rank‐based Kruskal–Wallis test (Kruskal and Wallis 1952) was used to assess differences in genetic diversity indices (*N*_A_, *A*_R_, *A*_P_, *H*_O_, *H*_E_, and *F*_IS_) across locations in R v. 4.4.1 (R Core Team 2024). When significant, pairwise comparisons were performed with Dunn's test (Dunn 1961) using the package ‘dunn.test’ v. 1.3.6 (Dinno 2024).

### 2.6 Population Genetic Structure and Migration Analysis

Population genetic structure was assessed using complementary approaches. Pairwise *F*_ST_ (Weir and ockerham 1984) and Jost's *D* values (Jost 2008) with 95% confidence intervals were estimated by bootstrapping (10,000 replicates) using the R package ‘diveRsity’ (Keenan et al. 2013). Jost's *D* was applied due to multi‐allelic markers, and *p*‐values were adjusted with a Bonferroni correction (Rice 1989). To assess the hierarchical structuring of microsatellite variation among groups, within groups, and within populations, analyses of molecular variance (AMOVA) were conducted with a distance matrix using the number of different alleles (*F*_ST_) and 20,000 permutations to test for statistical significance (Excoffier et al. 2005). Samples were grouped into two geographic clusters: Northern Hemisphere (BT, EE, BM, and BA) and Southern Hemisphere (AN, CH, CO, and JF).

A discriminant analysis of principal components (DAPC) was performed using the R package ‘adegenet’ v. 2.1.6 (Jombart 2008; Jombart et al. 2010) to visualize genotypic partitioning across: (1) the entire dataset of the Northern and Southern Hemisphere populations and (2) a partial dataset of the Temperate Northern Pacific populations. A genetic similarity dendrogram was constructed using Nei's genetic distance (*D*_a_) and the Unweighted Pair Group Method with Arithmetic Mean (UPGMA) method in the R package ‘mdendro’ v. 2.2.3 (Fernández and Gómez 2025), with the calculated cophenetic correlation coefficient (Sokal and Rohlf 1962). Finally, a Bayesian clustering analysis was conducted in STRUCTURE v. 2.3.4 (Pritchard et al. 2000) to examine population structure using a maximum likelihood approach under an admixture model with correlated allele frequencies and no prior population information. Initial rounds comprised 10 independent simulations from *k* = 1 to *k* = 14, each with 1000,000 Markov chain Monte Carlo repetitions and a 100,000‐step burn‐in period. Additional STRUCTURE rounds were run based on identified clusters to account for hierarchical genetic structure (Vähä et al. 2008) to reduce the confounding effects of substantial structure that could obscure finer‐scale differentiation. The most probable number of genetic clusters (*K*) was determined using the Evanno method based on a maximum likelihood framework (Evanno et al. 2005) implemented in STRUCTURE HARVESTER (Earl and VonHoldt 2012). Clustering results were visualized using CLUMPAK (Kopelman et al. 2015), which graphically represents individual assignment probabilities across inferred genetic clusters. To identify patterns of population genetic variation that derive from spatially limited gene flow, isolation‐by‐distance (IBD) was tested for Northern Hemisphere populations using Mantel tests between pairwise *F*_ST_ values and log‐transformed geographic distances (Rousset 1997). Pearson correlation coefficients and *p*‐values based on 20,000 permutations were calculated using Isolation by Distance Web Service v. 3.23 (Jensen et al. 2005).

To assess the magnitude and direction of gene flow among *S. lalandi* populations, we employed the directional relative migration method based on GST estimates (Sundqvist et al. 2016), implemented with the ‘divMigrate()’ function of the R package ‘diveRsity’ (Keenan et al. 2013). This approach estimates directional relative migration coefficients from allele‐frequency‐based genetic differentiation between pairs of populations, rather than direct demographic estimates of individual movement. Values range from 0 to 1, with higher values indicating stronger relative genetic connectivity in a given direction. Directional asymmetry was tested using 10,000 bootstrap replicates to generate 95% confidence intervals, identifying significantly biased gene flow between population pairs. Results were visualized using the R package ‘ggplot2’ (Wickham 2016).

## 3 Results

### 3.1 Genetic Diversity of Wild Populations and Captive‐Reared *Seriola lalandi* Stocks

To avoid overestimating genetic differentiation due to the Wahlund effect, 96 putative full‐sibs (relatedness > 0.45) were excluded from wild samples (Table S3). Additionally, 56 genotypes with null alleles at more than two loci and the *Sdu21* locus (12% missing data) were excluded. The final dataset included 497 individuals from 8 wild locations and 4 captive‐reared stocks.

All eight retained microsatellite loci were polymorphic in each of the 12 sampling populations from Temperate South America and the Temperate Northern Pacific (Table 1 and Table S4) and displayed high overall genetic variability. Among the wild populations, Northern Hemisphere fish exhibited higher expected heterozygosity than those from Chile in the Southern Hemisphere (Table 1). Samples from wild populations in Mexico (*H*_O_ = 0.733; *H*_E_ = 0.787) showed higher *H*_O_ and *H*_E_ values than those from Chile (*H*_O_ = 0.645; *H*_E_ = 0.715). The lowest allelic richness (*A*_R_ = 3.97) was observed in JF, whereas the highest was observed in BA (*A*_R_ = 5.01). In addition, CH showed the lowest heterozygosity (*H*_O_ = 0.631; *H*_E_ = 0.693), whereas BM and BA displayed the highest *H*_O_ (0.758) and *H*_E_ (0.803) values, respectively (Table 1). Among captive‐reared stocks, F1CL exhibited the lowest *A*_R_ (3.24), *H*_O_ (0.668), and *H*_E_ (0.615) values, while the highest were recorded in F1US (*A*_R_ = 4.33; *H*_O_ = 0.839; *H*_E_ = 0.751).

**TABLE 1:** Sampling locations and mean genetic diversity statistics calculated across the eight microsatellite loci included in the final dataset for wild and captive‐reared *Seriola lalandi* .

Origin	Country	Code	Collection site	*N*	*A*_R_	*A*_P_	*H*_O_	*H*_E_	*F*_IS_
Wild	Mexico	BT	Bahia de Todos Santos	16	4.87	2.74	0.719	0.783	0.135
Wild	Mexico	EE	Ejido Erendira	24	4.89	2.76	0.703	0.789	0.133
Wild	Mexico	BM	Bahia Magdalena	30	4.81	2.68	0.758	0.772	0.043
Wild	Mexico	BA	Bahia de los Angeles	45	5.01	2.90	0.750	0.803	0.069
Wild	Chile	AN	Antofagasta	35	4.33	1.80	0.642	0.747	0.159
Wild	Chile	CH	Chañaral	22	4.02	1.60	0.631	0.693	0.094
Wild	Chile	CO	Coquimbo	33	4.20	1.71	0.674	0.716	0.069
Wild	Chile	JF	Juan Fernandez	32	3.97	1.49	0.633	0.705	0.110
Captive‐reared	Mexico	F1BT	F1 Bahia de Todos Santos	96	4.18	2.23	0.727	0.733	0.009
Captive‐reared	Mexico	F1BM	F1 Bahia Magdalena	79	4.03	2.30	0.754	0.727	0.009
Captive‐reared	USA	F1US	F1 United States	39	4.33	2.45	0.839	0.751	−0.114
Captive‐reared	Chile	F1CL	F1 Chile	46	3.24	0.97	0.668	0.615	−0.085

Abbreviations: *A*_P_, private alleles; *A*_R_, allelic richness; *F*_IS_, inbreeding coefficient; *H*_E_, expected heterozygosity; *H*_O_, observed heterozygosity; *N*, Sample size.

Significant differences in *F*_IS_ (*p* = 0.000045, Kruskal–Wallis) were found between F1CL (*F*_IS_ = −0.085) and AN (*F*_IS_ = 0.159, *p* = 0.011, Dunn test) and EE (*F*_IS_ = 0.133, *p* = 0.021, Dunn test). Similarly, the *F*_IS_ of F1US differed significantly from that of EE (*p* = 0.006, Dunn test) and AN (*p* = 0.003, Dunn test) (Table 1 and Figure S1). Most loci conformed to HWE expectations across sampling locations. Deviations observed at one or two loci were not consistent across samples and were likely attributable to stochastic variation or homozygote excess (Table S2) rather than true departures from HWE at the population level (Table S4).

### 3.2 Genetic Population Structure of Wild and Captive‐Reared *Seriola lalandi*

Pairwise *F*_ST_ and Jost's *D* comparisons for microsatellites revealed significant inter‐hemispheric genetic divergence (*p* < 0.05), with *F*_ST_ ranging from 0.162 (BA vs. AN) to 0.208 (BM vs. CH), and Jost's *D* from 0.401 (JF vs. EE) to 0.565 (BT vs. AN; BM vs. CO) in wild samples (Table 2). At the regional scale, moderate but significant differentiation (*F*_ST_ = 0.078 and Jost's *D* = 0.111) was detected between wild fish of the most distant locations in Mexico, namely BT (Pacific coast) and BA (Gulf of California), even when atypical loci were excluded (Figure S2 and Table S5). In Chile, *S. lalandi* from AN to JF were largely panmictic (Table 2). Among hatchery stocks, significant genetic differences were detected in all pairwise comparisons between farmed and wild fish (Table 2). F1CL showed the highest divergence from wild BM (*F*_ST_ = 0.268; Jost's *D* = 0.703). In Chile, the lowest divergence was observed between F1CL and CH (*F*_ST_ = 0.021; Jost's *D* = 0.020). In the Temperate Northern Pacific, high divergence was observed between captive‐reared F1BM and wild BT (*F*_ST_ = 0.120; Jost's *D* = 0.212).

**TABLE 2:** Pairwise *F*_ST_ values (above diagonal) and Jost's *D* values (below diagonal) for microsatellite data.

	BT	EE	BM	BA	AN	CH	CO	JF	F1BT	F1BM	F1US	F1CL
BT		0.071*	0.030*	0.078*	0.176*	0.197*	0.195*	0.195*	0.089*	0.120*	0.118*	0.262*
EE	0.060		0.038*	−0.002	0.168*	0.192*	0.189*	0.186*	0.070*	0.079*	0.051*	0.252*
BM	0.023	0.028		0.043*	0.186*	0.208*	0.204*	0.202*	0.119*	0.105*	0.086*	0.268*
BA	0.111*	−0.001	0.028		0.162*	0.179*	0.181*	0.178*	0.065*	0.070*	0.051*	0.234*
AN	0.565*	0.443*	0.564*	0.464*		−0.002	−0.008	−0.002	0.211*	0.202*	0.199*	0.033*
CH	0.516*	0.456*	0.558*	0.448*	0.000		0.001	0.003	0.229*	0.223*	0.228*	0.021*
CO	0.525*	0.473*	0.565*	0.507*	−0.012	0.000		−0.006	0.227*	0.218*	0.217*	0.037*
JF	0.514*	0.401*	0.497*	0.456*	0.000	0.000	−0.003		0.227*	0.214*	0.217*	0.049*
F1BT	0.031	0.151*	0.190*	0.158*	0.639*	0.603*	0.622*	0.622*		0.069*	0.110*	0.276*
F1BM	0.212*	0.217*	0.173*	0.195*	0.605*	0.623*	0.640*	0.581*	0.163*		0.107*	0.273*
F1US	0.310*	0.150*	0.199*	0.163*	0.693*	0.729*	0.710*	0.652*	0.303*	0.292*		0.286*
F1CL	0.621*	0.603*	0.703*	0.599*	0.042*	0.020	0.049*	0.060*	0.664*	0.691*	0.851*	

*Note:* Locations (red markers) of wild *Seriola lalandi* populations: Bahia Todos Santos (BT), Ejido Erendira (EE), Bahia Magdalena (BM), Bahia de los Angeles (BA), Antofagasta (AN), Chañaral (CH), Coquimbo (CO), and Juan Fernandez (JF). Locations (red markers) of captive‐reared F1 *S. lalandi* stocks: Bahia Todos Santos (F1BT), Bahia Magdalena (F1BM), United States (F1US), and Chile (F1CL).

* Significant values (*p* < 0.05).

**FIGURE 2:**
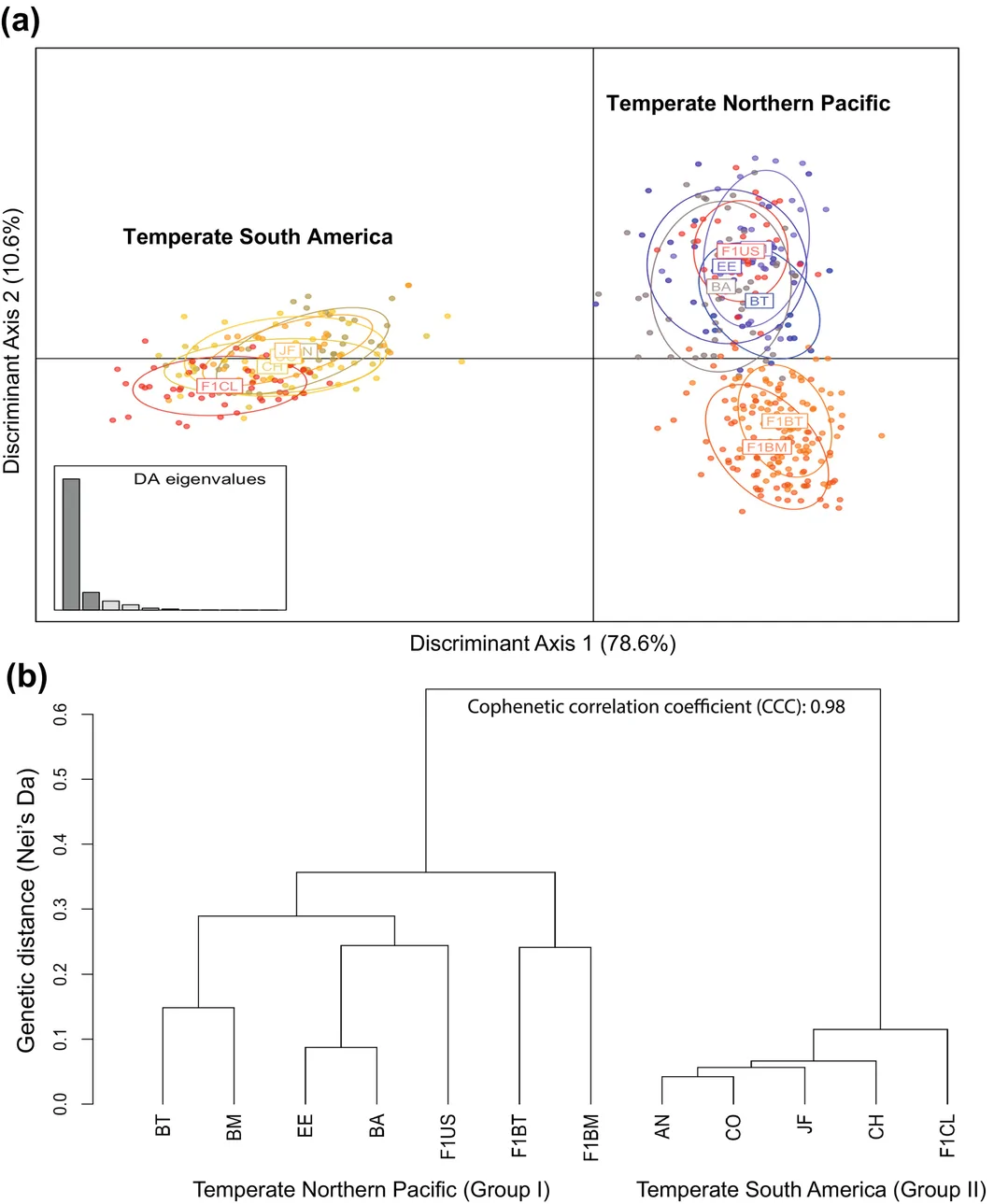
Discriminant Analysis of Principal Component (DAPC) and genetic similarity among *Seriola lalandi* captive‐reared and wild populations. (a) Scatterplots showing the first two principal components of the DAPC analysis. Clusters are shown by different colors of ellipses. Dots represent individuals. (b) Dendrogram for *S. lalandi* generated using Nei's D_a_ genetic distance and the UPGMA clustering method. Wild *S. lalandi* locations: Bahia Todos Santos (BT), Ejido Erendira (EE), Bahia Magdalena (BM), Bahia de los Angeles (BA), Antofagasta (AN), Chañaral (CH), Coquimbo (CO), and Juan Fernandez (JF). Captive‐reared *S. lalandi* stocks: Bahia Todos Santos (F1BT), Bahia Magdalena (F1BM), United States (F1US), and Chile (F1CL).

The AMOVA revealed that most genetic variation was found within populations (78%–90%), with limited variation among populations within groups (< 6%). High genetic differentiation was detected between hemispheres (*F*_CT_ = 0.1692, *p* < 0.001; Table S6), as well as moderate differentiation among populations within each hemisphere (*F*_SC_ = 0.0578, *p* < 0.001). The DAPC results (Figure 2a) clearly separated hemispheric populations, corresponding to the groups of the Temperate Northern Pacific and Temperate South America. Likewise, the UPGMA clustering results (Figure 2b) supported this grouping and identified two main groups with strong support based on the cophenetic correlation coefficient (0.98). Group I included all Temperate Northern Pacific populations (wild and captive‐reared from Mexico‐United States), with F1US clustering near BA and EE, and F1BT and F1BM forming a separate cluster from their respective wild origins (BT and BM). Group II encompassed the Chilean populations (wild and F1CL), with F1CL closely related to CH (Figure 2b).

**FIGURE 3:**
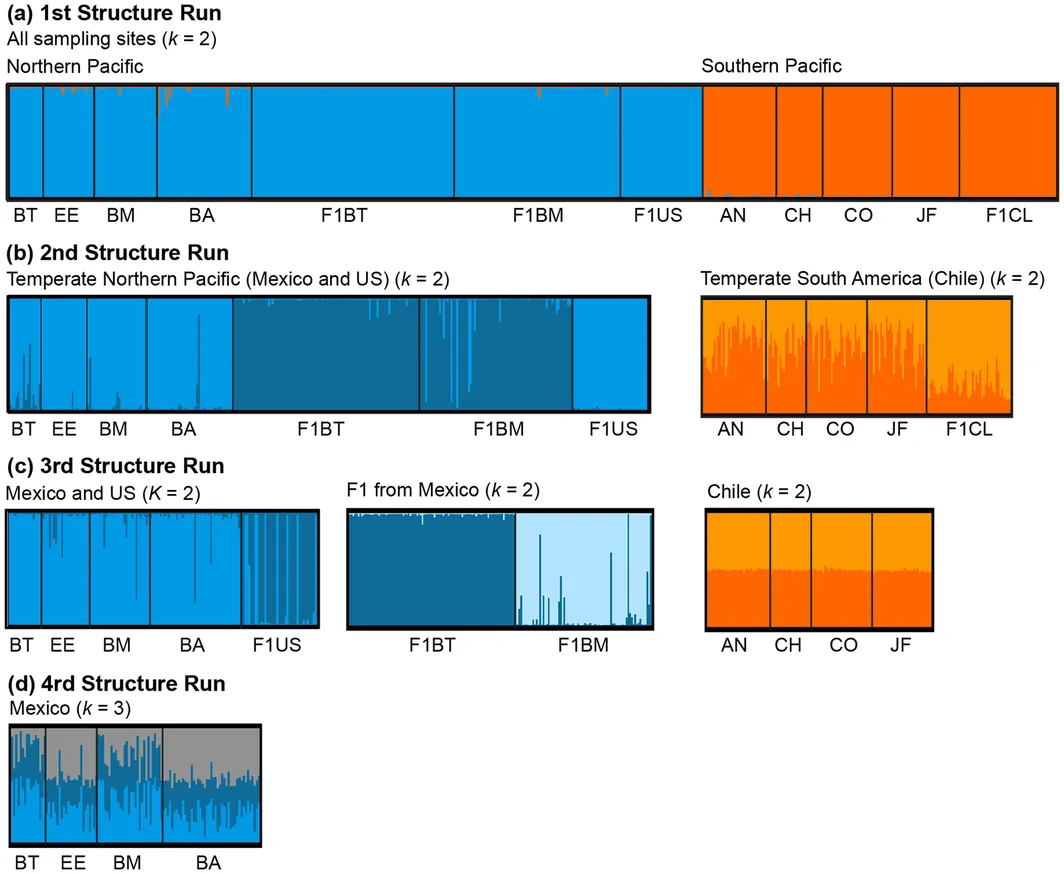
Hierarchical genetic structure of wild populations and captive‐reared stocks of *Seriola lalandi*. STRUCTURE bar plots show individual membership proportions across *K*‐inferred genetic clusters. Each vertical bar represents a single individual, and colors indicate the proportion of ancestry from each cluster. Panels represent successive hierarchical STRUCTURE analyses: (a) all sampling sites from the Northern and Southern Pacific (*K* = 2); (b) separate analyses of the Temperate Northern Pacific and Temperate South America (*K* = 2); (c) separate analyses of Mexico and the United States, F1 populations from Mexico, and Chile (*K* = 2); and (d) analysis restricted to wild Mexican populations (*K* = 3). Wild *S. lalandi* locations: Bahia Todos Santos (BT), Ejido Erendira (EE), Bahia Magdalena (BM), Bahia de los Angeles (BA), Antofagasta (AN), Chañaral (CH), Coquimbo (CO), and Juan Fernandez (JF). Captive‐reared *S. lalandi* stocks: Bahia Todos Santos (F1BT), Bahia Magdalena (F1BM), United States (F1US), and Chile (F1CL).

The first round of hierarchical STRUCTURE analysis, conducted without prior information, identified two main clusters corresponding to the Northern and Southern Hemispheres (Figure 3a and Figure S3). In the second round, separate analyses by hemisphere revealed genetic structure within the Temperate Northern Pacific, with F1BT and F1BM differing from BT, EE, BM, BA, and F1US. In the southern Pacific, subtle genetic differentiation was detected between wild individuals and captive‐reared stocks in Chile (Figure 3b). In the third round, analyses encompassing three groups revealed differentiation between the wild Mexican populations (BT, EE, BM, and BA) and the captive‐reared F1US stock of the United States, as well as between the captive‐reared stocks of F1BT and F1BM in Mexico (Figure 3c). No genetic differentiation was observed between wild populations in Chile. In the fourth round, separate analysis of wild Mexican populations revealed similarities between BT and BM and between EE and BA in Mexico (Figure 3d). Analysis of wild samples alone grouped populations into three clusters when *k* = 3: (1) BT and BM, (2) BA and EE, and (3) all Chilean sites (Figure S4). A Mantel test revealed no significant isolation by distance in the Northern Hemisphere (Figure S5).

**FIGURE 4:**
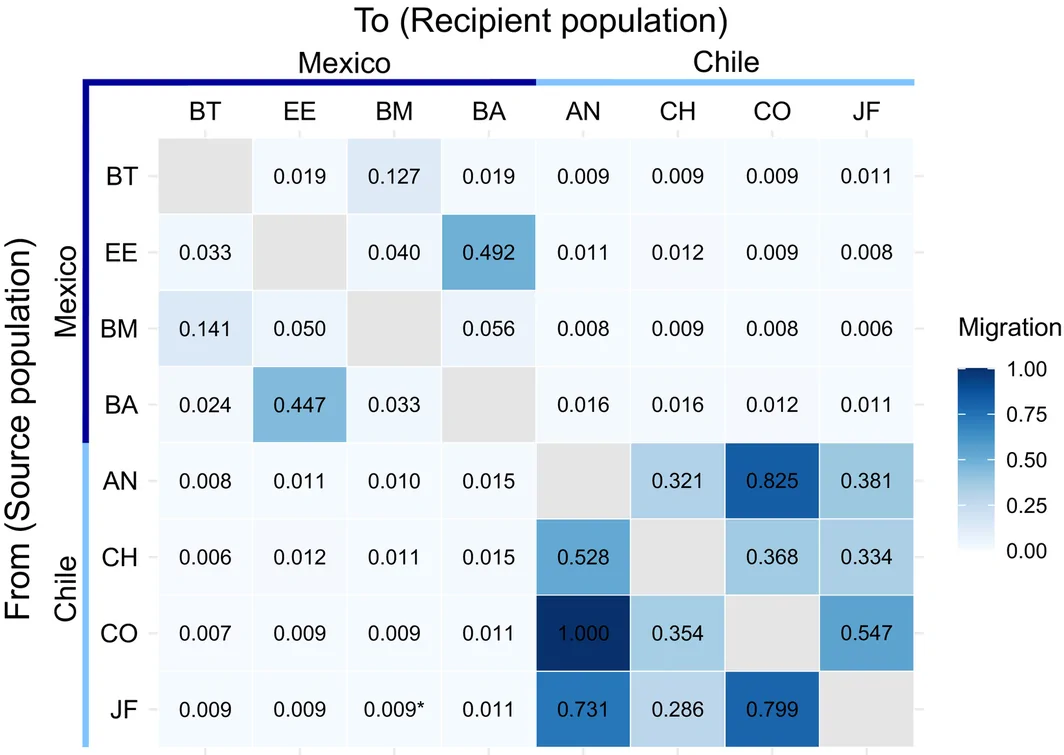
Pairwise relative directional migration matrix of *Seriola lalandi* from various locations in the Pacific Ocean. Rows indicate the source population (from), and columns indicate the recipient population (to). Values correspond to relative migration coefficients ranging from 0 to 1, with higher values indicating stronger relative genetic connectivity in a given direction. Values marked with an asterisk (*) indicate significant directional migration rates (*α* < 0.05).

Relative migration coefficients estimated using the ‘divMigrate()’ function (Keenan et al. 2013) revealed weak but statistically significant directional gene flow from Chile to Mexico (0.009; *α* < 0.05; Figure 4), predominantly toward BM. Within regions, bidirectional migration was consistently high. In the Temperate Northern Pacific, rates ranged from 0.019 to 0.492, with the highest rate observed between EE and BA. In Temperate South America, rates ranged from 0.286 to 1.00, with the strongest flow between CO and AN. However, these estimates were not statistically supported and should therefore be interpreted descriptively.

Together, our results confirm substantial genetic divergence between *S. lalandi* populations from the Northern and Southern Hemispheres, which corresponds to two well‐defined genetic groups identified across the Pacific Ocean: Temperate South America (Chile) and the Temperate Northern Pacific (Mexico). At the regional scale, moderate structure was also detected within the Northern Pacific locations between the Pacific coast (BT) and the Gulf of California (BA). All captive‐reared stocks were genetically distinct from wild populations, except F1BT, which was not significantly different from BT based on Jost's *D* values (Table 2).

## 4 Discussion

### 4.1 Global Population Structure of *Seriola lalandi*

The global genetic structure of *S. lalandi* is complex and comprises distinct population subdivisions in the Pacific Ocean (Martinez‐Takeshita et al. 2015; Purcell et al. 2015; Premachandra et al. 2017; Dettleff et al. 2020; Cui et al. 2023). Our study confirms strong hemispheric genetic divergence in *S. lalandi* that is likely driven by oceanographic barriers, thermal gradients, and differences in spawning seasons between the Northern and Southern Hemisphere populations (Sala et al. 2003; Moran et al. 2007; Pirozzi and Booth 2009). This pattern is observed in other widely distributed marine species, including the Tope shark ( *Galeorhinus galeus* ; Chabot and Allen 2009), Albacore ( *Thunnus alalunga* ; Vaux et al. 2021), Wreckfish ( *Polyprion americanus* ; Presa et al. 2023), and Yellowtail Amberjack ( *Seriola aureovittata* ; Cui et al. 2023).

Martinez‐Takeshita et al. (2015) proposed that only populations in the Southern Hemisphere correspond to the species *S. lalandi* , whereas those from the Temperate Northern Pacific (e.g., Mexico) should be reassigned and designated *Seriola dorsalis* Gill (1863). Despite the limited phylogenetic resolution of microsatellites, our results are consistent with previous evidence of strong divergence in this species (Purcell et al. 2015; Swart et al. 2016; Dettleff et al. 2020). Premachandra et al. (2017) used the same microsatellite panel applied in the present study, plus one additional locus, Sdu21, and reported an *F*_ST_ value of 0.200 between populations from Mexico and Chile. Our results are highly consistent with that estimate, as we observed similar levels of differentiation between Northern and Southern Hemisphere populations, including between Bahia de Todos Santos, Mexico, and Chañaral, Chile (*F*_ST_ = 0.197), and between Bahia Magdalena, Mexico, and Juan Fernandez, Chile (*F*_ST_ = 0.202).

Notably, Premachandra et al. (2017) reported that approximately 70% of SNPs (*F*_ST_ = 0.70) between Northern and Southern Hemisphere populations were differentially fixed, a remarkable level of divergence given that marine fishes typically exhibit lower differentiation (e.g., *F*_ST_ < 0.20; Gandra et al. 2021). Additional evidence includes differences in genome size between Mexican and Chilean *Seriola lalandi* (del Mar Ochoa‐Saloma et al. 2020) and phylogenomic analyses showing that *S. lalandi* represents the ancestral lineage from which *S. aureovittata* and *S. dorsalis* diverged approximately 3.8 Ma (Li et al. 2022). Together, these findings contribute to the growing body of evidence supporting the recognition of distinct species within the *Seriola lalandi* complex.

Studies in the Southern Hemisphere have demonstrated significant genetic differences within *S. lalandi* populations. For instance, Purcell et al. (2015) and Swart et al. (2016), established two distinct genetic lineages corresponding to the South Pacific and South African regions. Furthermore, Kerwath et al. (2021) proposed the existence of three genetically differentiated groups in southern Africa, highlighting a finer‐scale population structure in this region. Similarly, Premachandra et al. (2017) found that *S. lalandi* from Australia and Chile differed significantly based on microsatellite, mitochondrial DNA, SNP, and DArT markers (Premachandra et al. 2017). Nevertheless, the authors unexpectedly concluded that there were no genetic differences between *S. lalandi* from Australia and Chile, considering them a single population.

Together, our findings, along with those of earlier studies (Nugroho et al. 2001; Miller et al. 2011; Martinez‐Takeshita et al. 2015; Purcell et al. 2015; Swart et al. 2016; Sepúlveda and González 2017; Premachandra et al. 2017; Dettleff et al. 2020; Kerwath et al. 2021), demonstrate the existence of different genetic lineages within the recognized distribution of *S. lalandi sensu lato*, which inhabit the following marine regions (Spalding et al. 2007): Cold Temperate Northwest Pacific (Japan), Warm Temperate Northeast Pacific (Southern California Bight [Mexico‐United States] and Cortezian [Gulf of California, Mexico]), Warm Temperate Southeastern Pacific (Chile), Southeast Australian Shelf (New South Wales [Australia]), Southwest Australian Shelf (Geraldton [Western Australia]), South Atlantic (Vema seamount), Temperate Southern Africa (South Africa), and Western Indian Ocean (Walters Shoal seamount).

### 4.2 Population Differentiation and Stock Delineation Within Regions

Tagging experiments have shown that *S. lalandi* are able to travel up to 3000 km (Gillanders et al. 2001), although their migratory behavior generally occurs at regional scales (Baxter 1960; Miller et al. 2011). The long‐distance dispersal capacity inferred for *S. lalandi* aligns with our findings in the Temperate Northern Pacific, where individuals from the Mexico‐United States region correspond taxonomically to *S. dorsalis* according to Martinez‐Takeshita et al. (2015). We observed bidirectional migration between BA and EE (Figure 4), which are separated by ~2000 km. Our results reveal moderate genetic differentiation within the Temperate Northern Pacific, notably between BT and BA (*F*_ST_ = 0.078; Jost's *D* = 0.111). This divergence likely reflects ecological and oceanographic discontinuities within the Gulf of California, where the Midriff Islands and intense upwelling create a barrier separating the northern and central‐southern regions of the gulf (Thomson and Gilligan 2002). These findings indicate the presence of two ecologically distinct subpopulations of *S. dorsalis* in Mexican waters: one confined to the upper Gulf of California and another spanning the central–southern region of the Gulf of California and Pacific coast. Given the schooling behavior of this species, mating among related individuals within localized groups may also contribute to the maintenance of fine‐scale genetic structure (Fernández et al. 2015).

A similar pattern of genetic differentiation between the Gulf of California and Pacific Ocean has been reported in other coastal fishes, such as the Opaleye ( *Girella nigricans* ), California Grunion ( *Leuresthes tenuis* ), and Xantic Sargo ( *Anisotremus davidsonii* ), which has often been attributed to variations in pelagic larval dispersal, historical vicariance, and environmental conditions (e.g., salinity, temperature, and tidal dynamics) (Bernardi et al. 2003). More recent studies have documented comparable structuring in the Temperate Northern Pacific. For instance, the North Pacific Hake ( *Merluccius productus* ) exhibits genetic differentiation between populations in the northern Gulf of California and those along the Pacific coast (García‐De León et al. 2018). Similarly, de Jesús‐Bonilla et al. (2025) identified a clear north–south genetic break in the Panamic Redhead Goby ( *Elacatinus puncticulatus* ) within the Gulf of California, with individuals from Bahía de los Angeles genetically distinct from those in southern locations such as Los Cabos, La Paz, and Loreto. Notably, the regional population structure found in Mexico is similar to that found in South Africa (Swart et al. 2016; Kerwath et al. 2021), suggesting that *S. lalandi* may form distinct regional lineages across the Pacific and Atlantic basins. Future research, including broader sampling in the Gulf of California and the incorporation of additional molecular markers, is needed to confirm these differences.

Lower genetic diversity was observed among wild *S. lalandi* from Chile than among individuals from Mexico. This pattern may partly reflect regional differences in fisheries exploitation. In Chile, *S. lalandi* is a targeted fishery resource, with reported landings of approximately 482 tons in 2024 (SERNAPESCA 2024). In contrast, *S. dorsalis* has limited commercial importance in Mexico and is generally captured as bycatch (SADER 2023). No significant genetic structure was detected in *S. lalandi* populations in Chile, which suggests high gene flow and panmixia within this region. This finding also supports previous reports of genetic homogeneity along the continental coast and the offshore site of JF in Chile (Fernández et al. 2015), despite evidence of temporal variation (Sepúlveda and González 2017). Although JF is considered a biogeographic barrier in the southern Pacific (Reis et al. 2016), it appears to have little influence on the genetic structure of *S. lalandi* in this region.

### 4.3 Effects of Captivity on Genetic Composition and Implications for Aquaculture Management

This study revealed clear genetic divergence between captive‐reared stocks and wild populations of *S. lalandi* , which likely resulted from genetic drift arising from unequal reproductive contributions among broodstock (Knibb, Miller, et al. 2016; Schmidt et al. 2021; Dettleff et al. 2020). In a recent study, Cui et al. (2023) reported genetic divergence between wild *S. lalandi* from New South Wales, Australia, and F2 hatchery progeny, suggesting that detectable shifts in genetic structure can occur after only a few generations in captivity. This pattern may reflect the effects of captive breeding, as the genetic composition of the F2 cohort suggests a predominant contribution from a single breeding pair (Knibb, Elizur, et al. 2016). Such unequal parental contribution can reduce allelic diversity across generations, even when multiple F1 and F2 groups are pooled. Our findings align with evidence from other marine fishes, such as Atlantic Halibut ( *Hippoglossus hippoglossus* ) (Jackson et al. 2003) and Barramundi ( *Lates calcarifer* ) (Frost et al. 2006), where captive breeding has often lowered genetic diversity, which is likely attributable to unequal reproductive success among broodstock.

Sexual bias in spawning is frequently observed in *S. lalandi* and *S. dorsalis* hatcheries, with females contributing less to offspring than males. For example, Knibb, Miller, et al. (2016) reported that under controlled tank spawning conditions, 14%–67% of broodstock females and 80%–100% of broodstock males contributed genetically to offspring, depending on the tank. Dettleff et al. (2020) reported that male contributions were double those of females, and Schmidt et al. (2021) found that one female accounted for 40% of all progeny across two spawning seasons. These reproductive imbalances result in strong founder effects, reduced effective population size, and altered allele frequencies in hatchery offspring, which are probably attributable to selective mating behavior, gamete competition, or differential survival during initial life stages (Rodriguez‐Barreto et al. 2013; Kajino et al. 2025). This highlights the importance of conducting genetic monitoring and ensuring balanced mating schemes to preserve genetic diversity in cultured stocks. Additionally, photoperiod manipulation, hormonal induction, and cryopreservation techniques could support the preservation of the genetic variability necessary for the implementation of selective breeding programs (Shu‐Chien et al. 2025).

Chilean *S. lalandi* fingerlings are transported internationally for aquaculture, which raises important considerations regarding the genetic consequences of moving regionally differentiated stocks (Kolkovski and Lacámara 2014; Premachandra et al. 2017; Dettleff et al. 2020). The population subdivision reported in both the present and previous studies (Martinez‐Takeshita et al. 2015; Dettleff et al. 2020) should be carefully considered when translocating *S. lalandi* for aquaculture. This is particularly important when transferring fish between the Southern and Northern Hemispheres, or between Chilean and Australian stocks, to reduce the risk of disrupting local adaptation within the recipient populations. Furthermore, unregulated transport of fingerlings among regions may affect wild stocks through the unintended escape of genetically distinct individuals, as has been observed with the Atlantic Salmon ( *Salmo salar* ) (Wacker et al. 2021). Indeed, introgression from farmed *S. salar* escapees has been associated with delayed migratory behavior and reduced survival in wild populations (Bolstad et al. 2017; Wacker et al. 2021).

From an aquaculture perspective, genetic differentiation may represent a valuable resource for selective breeding. Approaches such as controlled hybridization and subsequent backcrossing could facilitate the incorporation of favorable traits from divergent parents to offspring, including enhanced growth, disease resistance, and stress tolerance (Liu et al. 2024). In China, hybrid lines account for approximately 32% of commercially authorized fish breeds (Liu et al. 2025), underscoring the practical relevance of such strategies.

In *Seriola*, natural hybridization between *S. quinqueradiata* and *S. lalandi* has been documented in Japan, and the presence of mature oocytes in F1 hybrid females, along with successful backcrosses, indicates partial fertility and reproductive compatibility (Takahashi et al. 2021). Natural hybridization may contribute to evolutionary novelty and, in some cases, adaptive variation; however, anthropogenically driven hybridization or introgression can threaten the genetic integrity of wild populations, particularly when non‐native, captive‐reared, or genetically differentiated stocks are released or escape into natural environments (Rhymer and Simberloff 1996; Simberloff 1996; Allendorf et al. 2001; Fitzpatrick et al. 2015). For example, Wringe et al. (2018) documented hybridization between domestic and wild Atlantic salmon following an accidental escape event, with hybrids representing approximately 27% of the sampled population. However, the proportion of individuals with domestic ancestry declined progressively over time relative to wild individuals, suggesting that introgression from domestic stocks may be associated with reduced survival or fitness in wild salmon populations.

Although controlled hybridization and genetic improvement strategies may have potential applications in aquaculture, their structured application in *Seriola* breeding programs remains largely unexplored and warrants further investigation under controlled conditions. Therefore, fish transfers or hybridization strategies should be guided by genetic compatibility, genetic monitoring, biosecurity protocols and supported by regulatory surveillance to ensure that aquaculture expansion does not compromise wild biodiversity.

## 5 Conclusions

We confirmed hemispheric genetic differentiation in *S. lalandi* , consistent with previous evidence of divergent lineages. Moderate genetic differentiation was also evident within the Temperate Northern Pacific (BT vs. BA), whereas populations within Chile appeared largely panmictic, suggesting that broader environmental and oceanographic factors may influence fine‐scale genetic structure in the Pacific Ocean. Additionally, genetic differences between wild and captive‐reared individuals were detected, likely reflecting skewed broodstock contributions.

Given the global demand for *S. lalandi* , future research should prioritize studies on reproductive management in captivity and evaluate key aquaculture traits, such as growth rates and disease resistance, across genetically distinct populations. Moreover, given the economic and ecological importance of *S. lalandi* , robust phylogeographic and taxonomic studies using sequence‐based molecular markers, along with reproductive research, are essential for conserving genetic diversity and guiding local fisheries management and international aquaculture practices.

## Author Contributions

**Eduardo Martínez‐Matus:** conceptualization (equal), data curation (equal), formal analysis (equal), investigation (equal), methodology (equal), software (equal), visualization (equal), writing – original draft (equal). **Felipe Aguilera:** formal analysis (equal), investigation (equal), methodology (equal), validation (equal), writing – original draft (equal), writing – review and editing (equal). **Raquel Muñiz‐Salazar:** formal analysis (equal), validation (equal), writing – review and editing (equal). **Fabiola A. Sepúlveda:** resources (equal), writing – review and editing (equal). **M. Teresa González:** funding acquisition (supporting), resources (equal), writing – review and editing (equal). **Cristian Araneda‐Tolosa:** formal analysis (equal), validation (equal), writing – review and editing (equal). **Claudia Farfán:** funding acquisition (equal), investigation (equal), resources (equal), supervision (equal), validation (equal), writing – original draft (equal), writing – review and editing (equal). **Fabiola Lafarga‐De la Cruz:** conceptualization (equal), funding acquisition (lead), investigation (lead), project administration (lead), resources (lead), supervision (lead), validation (equal), writing – original draft (equal), writing – review and editing (lead).

## Funding

This work was supported by Secretaría de Agricultura, Ganadería, Desarrollo Rural, Pesca y Alimentación, INAPESCA 2016 Genetic Aquatic Resources 2505160166. Consejo Nacional de Ciencia y Tecnología, Mexico, Scholarship 394575. Agencia Nacional de Investigación y Desarrollo, 21232432.

## Conflicts of Interest

The authors declare no conflicts of interest.

## Supporting information


**Table S1:** Sampling sites and number of *Seriola lalandi* individuals collected and analyzed in this study.
**Table S2:** Null allele frequencies for each locus and location sampled. Locations of wild *Seriola lalandi* : Bahia Todos Santos (BT), Ejido Erendira (EE), Bahia Magdalena (BM), Bahia de los Angeles (BA), Antofagasta (AN), Chañaral (CH), Coquimbo (CO), Juan Fernandez (JF). Locations of captive‐reared *S. lalandi* : Bahia Todos Santos (F1BT), Bahia Magdalena (F1BM), United States (F1US), and Chile (F1CL).
**Table S3:** Mean relatedness *r* (±SE) and relationship percentage for all sampling sites. Locations of wild *Seriola lalandi* : Bahia Todos Santos (BT), Ejido Erendira (EE), Bahia Magdalena (BM), Bahia de los Angeles (BA), Antofagasta (AN), Chañaral (CH), Coquimbo (CO), Juan Fernandez (JF). Locations of captive‐reared *S. lalandi* : Bahia Todos Santos (F1BT), Bahia Magdalena (F1BM), United States (F1US), and Chile (F1CL).
**Table S4:** Genetic diversity parameters per locus of wild and captive‐reared *Seriola lalandi* . *N*: sample size, *N*_A_: allele number, *A*_R_: allelic richness, *H*_O_: observed heterozygosity, *H*_E_: expected heterozygosity, HWE: Hardy–Weinberg equilibrium.
**Table S5:** Pairwise *F*_ST_ (above the diagonal) and Jost's *D* values (below the diagonal) calculated after excluding loci *Sdu32* and *Sdu46*. Only values highlighted in red were significant when using the full 8‐locus dataset. Asterisks (*) denote significance at *p* < 0.05 based on 95% confidence intervals with 10,000 bootstrap replicates.
**Table S6:** Analysis of molecular variance (AMOVA) of *Seriola lalandi* in the Pacific.
**Figure S1:** Inbreeding coefficients (*F*_IS_) of 10 groups of *Seriola lalandi* from the northern and southern Pacific. Boxplots represent the median and interquartile ranges of *F*_IS_ values for wild and captive‐reared *S. lalandi* , labeled as in Figure 1. Boxplots sharing the same letter differ significantly (*p* < 0.05) according to Dunn's test with Bonferroni correction.
**Figure S2:** Identification of loci potentially under selection. Loci *Sdu46* and *Sdu32*, initially developed for *Seriola dumerili* (Renshaw et al. 2007), were identified as outliers using a false discovery rate (FDR) threshold of < 0.001.
**Figure S3:** ΔK values calculated using the Evanno method (Evanno et al. 2005) for the main STRUCTURE analyses: (a) all locations from both hemispheres, (b) Temperate Northern Pacific locations only, (c) locations of wild *Seriola lalandi* in Mexico, and (d) Chile. Plots indicate the most likely number of genetic clusters in each group.
**Figure S4:** (a) DAPC of 8 wild *Seriola lalandi* sampling locations showing distinct clusters corresponding to the Temperate South America, and Temperate Northern Pacific. Notably, fish from Bahia de Todos Santos (BT) are separate from those in Bahia de Los Angeles (BA) and Ejido Erendira (EE). (b) STRUCTURE analysis of 8 wild populations. At *k* = 3, clear genetic differentiation is observed among Mexican populations, while Chilean samples appear genetically homogeneous.
**Figure S5:** Scatterplot of the isolation‐by‐distance (IBD) analysis among *Seriola lalandi* samples. The Mantel's test for correlation between *F*_ST_ and log‐transformed geographic distance for Temperate Northern Pacific.

## Data Availability

Microsatellite genotypes for all Yellowtail kingfish ( *Seriola lalandi* ) samples analyzed in this study are available from the DRYAD repository: https://doi.org/10.5061/dryad.4xgxd25p4.
