# Supplementary material for: Population Genetics of Seriola lalandi Confirm Hemispheric Divergence and Reveal Genetic Structure in the Temperate Northern Pacific and Between Wild and Captive‐Reared Fish
Source: Ecol Evol. 2026 Aug 20;16(8):e74200. doi: 10.1002/ece3.74200 (PMC13491388; doi:10.1002/ece3.74200)
Supplement: Supplementary file 1 [file ECE3-16-e74200-s001.docx]

**Supplementary Material**

Population genetics of *Seriola lalandi* confirm hemispheric divergence and reveal genetic structure in the Temperate Northern Pacific and between wild and captive-reared fish

Eduardo Martínez-Matus^1,2†^, Felipe Aguilera^3†^, Raquel Muñiz-Salazar^4^, Fabiola A. Sepúlveda^5^, M. Teresa González^5^, Cristian Araneda-Tolosa^6^, Claudia Farfán^1‡*^, Fabiola Lafarga-De la Cruz^1^*

^1^Departamento de Acuicultura, División de Oceanología, Centro de Investigación Científica y de Educación Superior de Ensenada (CICESE), Ensenada, Baja California, México.

^2^Instituto de Biología, Facultad de Ciencias, Pontificia Universidad Católica de Valparaíso, Valparaíso, Chile.

^3^Departamento de Bioquímica y Biología Molecular, Facultad de Ciencias Biológicas, Universidad de Concepción, Barrio Universitario s/n, Casilla 160-C, Concepción, Chile.

^4^Laboratorio de Epidemiología y Ecología Molecular, Escuela Ciencias de la Salud, Universidad Autónoma de Baja California, Ensenada, Baja California, México.

^5^Instituto de Ciencias Naturales Alexander von Humboldt, Facultad de Ciencias del Mar y Recursos Biológicos, Universidad de Antofagasta, Antofagasta, Chile.

^6^Departamento de Producción Animal, Facultad de Ciencias Agronómicas, Universidad de Chile, Avda. Santa Rosa 11315, La Pintana 8820808, Santiago, Chile.

^†^These authors contributed equally to this work.

^‡^Deceased.

**Co-corresponding authors**: Fabiola Lafarga-De la Cruz. Email: [flafarga@cicese.edu.mx](mailto:flafarga@cicese.edu.mx); Claudia Farfán^1‡^

**Keywords:** genetic diversity, genetic structure, yellowtail kingfish, genetic conservation, broodstock management, aquaculture

**Table S1.** Sampling sites and number of *Seriola lalandi* individuals collected and analyzed in this study.

|  | | **Wild fish** | | | | |
| --- | --- | --- | --- | --- | --- | --- |
| na | nb | | Sampling Site | Region | Longitude | Latitude |
| 17 | 16 | | Bahia de Todos Santos, Mexico (BT) | Temperate Northern Pacific | –116.800634 | 31.738566 |
| 24 | 24 | | Ejido Erendira, Mexico (EE) | Temperate Northern Pacific | –116.4948167 | 31.2267556 |
| 30 | 30 | | Bahia Magdalena, Mexico (BM) | Temperate Northern Pacific | –112.0670111 | 24.3720167 |
| 45 | 45 | | Bahia de los Angeles, Mexico (BA) | Temperate Northern Pacific | –113.3072167 | 28.9453444 |
| 79 | 35 | | Antofagasta, Chile (AN) | Temperate South America | –70.486647 | –23.648396 |
| 28 | 22 | | Chañaral, Chile (CH) | Temperate South America | –70.93426944 | –26.1575833 |
| 53 | 33 | | Coquimbo, Chile (CO) | Temperate South America | –71.56489167 | –30.0408472 |
| 60 | 32 | | Juan Fernandez, Chile (JF) | Temperate South America | –78.915044 | –33.658286 |
|  | | **Captive-reared stocks** | | | | |
| na | nb | | Sampling site | Region | Hatchery | |
| 40 | 39 | | United States (F1US) | Temperate Northern Pacific | Hubbs Sea World Research Institute | |
| 125 | 96 | | Bahia de Todos Santos, Mexico (F1BT) | Temperate Northern Pacific | Baja Seas Aquaculture | |
| 98 | 79 | | Bahia Magdalena, Mexico (F1BM) | Temperate Northern Pacific | Baja Seas Aquaculture | |
| 50 | 46 | | Chile (F1CL) | Temperate South America | Acuinor | |

na: Number of genotyped individuals.

nb: Number of genotypes after correction for missing data and relatedness.

**Table S2.** Null allele frequencies for each locus and location sampled. Locations of wild *Seriola lalandi*: Bahia Todos Santos (BT), Ejido Erendira (EE), Bahia Magdalena (BM), Bahia de los Angeles (BA), Antofagasta (AN), Chañaral (CH), Coquimbo (CO), Juan Fernandez (JF). Locations of captive-reared *S. lalandi*: Bahia Todos Santos (F1BT), Bahia Magdalena (F1BM), United States (F1US), and Chile (F1CL).

| **Site** | ***Sel001*** | ***Sel002*** | ***Sel008*** | ***Sel011*** | ***Sel017*** | ***Sel019*** | ***Sdu32*** | ***Sdu46*** |
| --- | --- | --- | --- | --- | --- | --- | --- | --- |
| BT | 0.000 | 0.036 | 0.032 | 0.000 | 0.000 | 0.049 | 0.000 | 0.327 |
| EE | 0.040 | 0.026 | 0.065 | 0.016 | 0.044 | 0.000 | 0.085 | 0.096 |
| BM | 0.000 | 0.004 | 0.000 | 0.000 | 0.015 | 0.000 | 0.054 | 0.047 |
| BA | 0.040 | 0.000 | 0.019 | 0.030 | 0.034 | 0.000 | 0.072 | 0.000 |
| AN | 0.072 | 0.147 | 0.067 | 0.009 | 0.037 | 0.117 | 0.019 | 0.019 |
| CH | 0.052 | 0.124 | 0.000 | 0.000 | 0.000 | 0.000 | 0.147 | 0.000 |
| CO | 0.045 | 0.043 | 0.000 | 0.078 | 0.023 | 0.079 | 0.000 | 0.000 |
| JF | 0.069 | 0.094 | 0.000 | 0.000 | 0.000 | 0.103 | 0.142 | 0.064 |
| F1US | 0.000 | 0.000 | 0.000 | 0.000 | 0.000 | 0.000 | 0.000 | 0.000 |
| F1BT | 0.000 | 0.000 | 0.000 | 0.000 | 0.021 | 0.000 | 0.000 | 0.047 |
| F1BM | 0.000 | 0.000 | 0.000 | 0.000 | 0.064 | 0.034 | 0.045 | 0.000 |
| F1CL | 0.008 | 0.005 | 0.000 | 0.000 | 0.000 | 0.000 | 0.000 | 0.000 |

**Table S3.** Mean relatedness *r* (±SE) and relationship percentage for all sampling sites. Locations of wild *Seriola lalandi*: Bahia Todos Santos (BT), Ejido Erendira (EE), Bahia Magdalena (BM), Bahia de los Angeles (BA), Antofagasta (AN), Chañaral (CH), Coquimbo (CO), Juan Fernandez (JF). Locations of captive-reared *S. lalandi*: Bahia Todos Santos (F1BT), Bahia Magdalena (F1BM), United States (F1US), and Chile (F1CL).

| **Origin site** | ***r* (±SE)** | **Relatedness** | | |
| --- | --- | --- | --- | --- |
|  |  | **Unrelated** | **Half-sibs** | **Full-sibs** |
| BT | 0.031 ± 0.004 | 94.85% | 4.41% | 0.74% |
| EE | 0.029 ± 0.003 | 95.65% | 3.99% | 0.36% |
| BM | 0.038 ± 0.002 | 89.20% | 10.34% | 0.46% |
| BA | 0.039 ± 0.002 | 90.00% | 9.49% | 0.51% |
| AN | 0.064 ± 0.001 | 84.18% | 12.98% | 2.84% |
| CH | 0.049 ± 0.003 | 87.30% | 10.58% | 2.12% |
| CO | 0.059 ± 0.002 | 84.62% | 12.92% | 2.47% |
| JF | 0.064 ± 0.002 | 82.82% | 14.46% | 2.71% |
| F1US | 0.095 ± 0.004 | 78.00% | 9.72% | 12.28% |
| F1BT | 0.088 ± 0.002 | 78.62% | 14.12% | 7.26% |
| F1BM | 0.105 ± 0.002 | 76.73% | 10.61% | 12.66% |
| F1CL | 0.101 ± 0.004 | 77.39% | 12.85% | 9.76% |

**Table S4.** Genetic diversity parameters per locus of wild and captive-reared *Seriola lalandi*. *N*: sample size, *N*_A_: allele number, *A*_R_: allelic richness, *H*_O_: observed heterozygosity, *H*_E_: expected heterozygosity, HWE: Hardy-Weinberg equilibrium.

| **Wild locations** | ***N*** | **Parameter** | ***Sel001*** | ***Sel002*** | ***Sel008*** | ***Sel011*** | ***Sel017*** | ***Sel019*** | ***Sdu32*** | ***Sdu46*** | **Mean** |
| --- | --- | --- | --- | --- | --- | --- | --- | --- | --- | --- | --- |
| Bahia de Todos Santos, Mexico (BT) | 16 | *N*_A_ | 8 | 5 | 12 | 11 | 19 | 6 | 14 | 4 | 9.9 |
|  |  | *A*_R_ | 4.77 | 3.07 | 5.36 | 5.54 | 7.00 | 4.17 | 5.87 | 3.15 | 4.87 |
|  |  | *A*_P_ | 1.94 | 2.27 | 3.35 | 2.95 | 5.71 | 1.09 | 3.44 | 1.20 | 2.74 |
|  |  | *H*_O_ | 0.938 | 0.438 | 0.813 | 0.938 | 0.938 | 0.688 | 0.875 | 0.125 | 0.719 |
|  |  | *H*_E_ | 0.809 | 0.531 | 0.834 | 0.855 | 0.932 | 0.758 | 0.873 | 0.676 | 0.783 |
|  |  | *F*_IS_ | -0.128 | 0.208 | 0.058 | -0.064 | 0.026 | 0.125 | 0.030 | 0.826 | 0.135 |
|  |  | HWE | ns | ns | ns | ns | ns | ns | ns | *** |  |
| Ejido Erendira, Mexico (EE) | 24 | *N*_A_ | 10 | 4 | 12 | 13 | 24 | 7 | 15 | 7 | 11.5 |
|  |  | *A*_R_ | 5.02 | 2.58 | 5.39 | 5.39 | 6.88 | 4.29 | 6.14 | 3.40 | 4.89 |
|  |  | *A*_P_ | 2.46 | 1.12 | 3.42 | 2.84 | 5.60 | 1.25 | 3.59 | 1.77 | 2.76 |
|  |  | *H*_O_ | 0.750 | 0.500 | 0.750 | 0.833 | 0.833 | 0.750 | 0.750 | 0.458 | 0.703 |
|  |  | *H*_E_ | 0.836 | 0.568 | 0.860 | 0.854 | 0.934 | 0.777 | 0.903 | 0.578 | 0.789 |
|  |  | *F*_IS_ | 0.124 | 0.140 | 0.149 | 0.046 | 0.129 | 0.056 | 0.190 | 0.227 | 0.133 |
|  |  | HWE | ns | ns | ns | ns | ns | ns | ns | ns |  |
| Bahia Magdalena, Mexico (BM) | 30 | *N*_A_ | 9 | 5 | 18 | 11 | 24 | 9 | 18 | 7 | 12.6 |
|  |  | *A*_R_ | 4.36 | 3.35 | 6.01 | 5.27 | 6.68 | 4.66 | 5.85 | 2.31 | 4.81 |
|  |  | *A*_P_ | 1.61 | 2.39 | 3.83 | 2.69 | 5.22 | 1.52 | 3.38 | 0.81 | 2.68 |
|  |  | *H*_O_ | 0.867 | 0.700 | 0.867 | 0.900 | 0.867 | 0.833 | 0.767 | 0.267 | 0.758 |
|  |  | *H*_E_ | 0.788 | 0.682 | 0.896 | 0.853 | 0.929 | 0.818 | 0.887 | 0.324 | 0.772 |
|  |  | *F*_IS_ | -0.083 | -0.009 | 0.050 | -0.038 | 0.084 | -0.002 | 0.152 | 0.193 | 0.043 |
|  |  | HWE | ns | ns | ns | ns | ns | ns | ns | ** |  |
| Bahia de los Angeles, Mexico (BA) | 45 | *N*_A_ | 10 | 5 | 18 | 14 | 34 | 15 | 16 | 7 | 14.9 |
|  |  | *A*_R_ | 4.64 | 3.26 | 5.68 | 5.18 | 6.85 | 5.29 | 6.03 | 3.17 | 5.01 |
|  |  | *A*_P_ | 1.84 | 1.74 | 3.82 | 2.71 | 5.57 | 2.32 | 3.64 | 1.59 | 2.90 |
|  |  | *H*_O_ | 0.711 | 0.644 | 0.822 | 0.800 | 0.889 | 0.778 | 0.778 | 0.578 | 0.750 |
|  |  | *H*_E_ | 0.814 | 0.646 | 0.874 | 0.844 | 0.941 | 0.860 | 0.905 | 0.542 | 0.803 |
|  |  | *F*_IS_ | 0.137 | 0.013 | 0.071 | 0.063 | 0.066 | 0.107 | 0.152 | -0.054 | 0.069 |
|  |  | HWE | ns | ns | ns | ns | ns | ns | *** | ns |  |
| Antofagasta, Chile (AN) | 35 | *N*_A_ | 10 | 6 | 6 | 9 | 14 | 10 | 15 | 6 | 9.5 |
|  |  | *A*_R_ | 4.66 | 3.32 | 3.06 | 4.51 | 5.03 | 4.79 | 5.96 | 3.34 | 4.33 |
|  |  | *A*_P_ | 1.79 | 1.07 | 1.29 | 1.88 | 2.75 | 1.82 | 3.17 | 0.68 | 1.80 |
|  |  | *H*_O_ | 0.706 | 0.429 | 0.457 | 0.743 | 0.714 | 0.600 | 0.857 | 0.629 | 0.642 |
|  |  | *H*_E_ | 0.805 | 0.641 | 0.522 | 0.790 | 0.807 | 0.823 | 0.899 | 0.687 | 0.747 |
|  |  | *F*_IS_ | 0.138 | 0.344 | 0.138 | 0.074 | 0.130 | 0.284 | 0.061 | 0.099 | 0.159 |
|  |  | HWE | *** | *** | ns | ns | ns | ns | ns | ns |  |
| Chañaral, Chile (CH) | 22 | *N*_A_ | 9 | 4 | 5 | 8 | 13 | 6 | 14 | 7 | 8.3 |
|  |  | *A*_R_ | 4.28 | 2.66 | 2.63 | 3.82 | 4.60 | 4.34 | 6.37 | 3.45 | 4.02 |
|  |  | *A*_P_ | 1.51 | 0.52 | 0.97 | 1.45 | 2.58 | 1.34 | 3.58 | 0.84 | 1.60 |
|  |  | *H*_O_ | 0.591 | 0.409 | 0.500 | 0.636 | 0.773 | 0.818 | 0.636 | 0.682 | 0.631 |
|  |  | *H*_E_ | 0.758 | 0.579 | 0.417 | 0.706 | 0.724 | 0.783 | 0.913 | 0.663 | 0.693 |
|  |  | *F*_IS_ | 0.243 | 0.314 | -0.176 | 0.121 | -0.044 | -0.022 | 0.324 | -0.005 | 0.094 |
|  |  | HWE | ns | ns | ns | ns | ns | ns | ns | ns |  |
| Coquimbo, Chile (CO) | 33 | *N*_A_ | 12 | 5 | 7 | 10 | 15 | 7 | 13 | 6 | 9.4 |
|  |  | *A*_R_ | 4.72 | 2.89 | 2.82 | 4.37 | 5.00 | 4.49 | 6.01 | 3.27 | 4.20 |
|  |  | *A*_P_ | 1.92 | 0.75 | 1.15 | 1.81 | 2.69 | 1.49 | 3.23 | 0.65 | 1.71 |
|  |  | *H*_O_ | 0.727 | 0.485 | 0.455 | 0.667 | 0.788 | 0.667 | 0.879 | 0.727 | 0.674 |
|  |  | *H*_E_ | 0.804 | 0.528 | 0.453 | 0.758 | 0.802 | 0.805 | 0.901 | 0.675 | 0.716 |
|  |  | *F*_IS_ | 0.111 | 0.097 | 0.011 | 0.135 | 0.033 | 0.187 | 0.040 | -0.062 | 0.069 |
|  |  | HWE | ns | ns | ns | * | ns | ns | ns | ns |  |
| Juan Fernandez, Chile (JF) | 32 | *N*_A_ | 9 | 3 | 5 | 6 | 9 | 9 | 17 | 5 | 7.9 |
|  |  | *A*_R_ | 4.26 | 2.52 | 3.00 | 3.67 | 4.12 | 4.59 | 6.31 | 3.30 | 3.97 |
|  |  | *A*_P_ | 1.38 | 0.36 | 1.22 | 1.15 | 1.83 | 1.58 | 3.77 | 0.63 | 1.49 |
|  |  | *H*_O_ | 0.625 | 0.375 | 0.531 | 0.781 | 0.875 | 0.656 | 0.656 | 0.563 | 0.633 |
|  |  | *H*_E_ | 0.777 | 0.502 | 0.518 | 0.696 | 0.731 | 0.810 | 0.914 | 0.689 | 0.705 |
|  |  | *F*_IS_ | 0.211 | 0.268 | -0.010 | -0.107 | -0.182 | 0.205 | 0.296 | 0.199 | 0.110 |
|  |  | HWE | ns | ns | ns | ns | ns | ns | ** | ns |  |
| **Cultured stocks** | ***N*** | **Parameter** | ***Sel001*** | ***Sel002*** | ***Sel008*** | ***Sel011*** | ***Sel017*** | ***Sel019*** | ***Sdu32*** | ***Sdu46*** | **Mean** |
| Bahia de Todos Santos (F1BT) |  | *N*_A_ | 7 | 4 | 12 | 11 | 17 | 6 | 12 | 4 | 9.1 |
|  |  | *A*_R_ | 4.21 | 2.17 | 4.54 | 4.84 | 5.65 | 3.93 | 5.21 | 2.88 | 4.18 |
|  |  | *A*_P_ | 1.55 | 1.21 | 2.69 | 2.54 | 4.97 | 0.96 | 2.95 | 1.00 | 2.23 |
|  | 96 | *H*_O_ | 0.823 | 0.333 | 0.823 | 0.813 | 0.821 | 0.811 | 0.823 | 0.573 | 0.727 |
|  |  | *H*_E_ | 0.786 | 0.319 | 0.784 | 0.829 | 0.887 | 0.752 | 0.858 | 0.648 | 0.733 |
|  |  | *F*_IS_ | -0.042 | -0.040 | -0.045 | 0.025 | 0.079 | -0.073 | 0.046 | 0.121 | 0.009 |
|  |  | HWE | ns | ns | ns | ns | ns | ns | ns | ns |  |
| Bahia Magdalena (F1BM) |  | *N*_A_ | 8 | 4 | 14 | 9 | 17 | 8 | 13 | 6 | 9.9 |
|  |  | *A*_R_ | 3.81 | 2.23 | 4.73 | 4.23 | 4.87 | 4.52 | 5.40 | 2.44 | 4.03 |
|  |  | *A*_P_ | 1.14 | 1.17 | 3.08 | 2.57 | 4.38 | 1.75 | 3.60 | 0.69 | 2.30 |
|  | 79 | *H*_O_ | 0.797 | 0.494 | 1.000 | 0.861 | 0.759 | 0.743 | 0.823 | 0.551 | 0.754 |
|  |  | *H*_E_ | 0.744 | 0.473 | 0.805 | 0.776 | 0.817 | 0.812 | 0.876 | 0.513 | 0.727 |
|  |  | *F*_IS_ | -0.066 | -0.036 | -0.236 | -0.104 | 0.077 | 0.092 | 0.067 | -0.068 | -0.034 |
|  |  | HWE | ns | ns | *** | ns | *** | ns | *** | ns |  |
| United States (F1US) |  | *N*_A_ | 7 | 5 | 10 | 10 | 18 | 6 | 13 | 6 | 9.4 |
|  |  | *A*_R_ | 3.17 | 3.46 | 4.79 | 4.95 | 5.80 | 4.17 | 5.18 | 3.11 | 4.33 |
|  |  | *A*_P_ | 1.20 | 1.86 | 3.35 | 2.80 | 4.79 | 1.10 | 3.04 | 1.48 | 2.45 |
|  | 39 | *H*_O_ | 0.744 | 0.795 | 0.895 | 0.923 | 0.947 | 0.795 | 0.923 | 0.692 | 0.839 |
|  |  | *H*_E_ | 0.575 | 0.671 | 0.823 | 0.831 | 0.884 | 0.778 | 0.852 | 0.596 | 0.751 |
|  |  | *F*_IS_ | -0.281 | -0.172 | -0.074 | -0.098 | -0.058 | -0.009 | -0.071 | -0.148 | -0.114 |
|  |  | HWE | ns | ns | ns | ns | ns | ns | ns | ns |  |
| Chile (F1CL) |  | *N*_A_ | 8 | 3 | 5 | 5 | 5 | 5 | 11 | 5 | 5.9 |
|  |  | *A*_R_ | 3.86 | 2.56 | 2.10 | 3.59 | 2.89 | 2.89 | 4.86 | 3.21 | 3.24 |
|  |  | *A*_P_ | 1.07 | 0.36 | 0.65 | 1.07 | 0.90 | 0.54 | 2.49 | 0.64 | 0.97 |
|  | 46 | *H*_O_ | 0.717 | 0.609 | 0.326 | 0.783 | 0.630 | 0.717 | 0.870 | 0.696 | 0.668 |
|  |  | *H*_E_ | 0.741 | 0.579 | 0.290 | 0.721 | 0.512 | 0.622 | 0.824 | 0.635 | 0.615 |
|  |  | *F*_IS_ | 0.043 | -0.041 | -0.115 | -0.075 | -0.221 | -0.143 | -0.044 | -0.085 | -0.085 |
|  |  | HWE | ns | ns | ns | ns | ns | ns | ns | ns |  |

ns: not significant; *p < 0.05; **p < 0.01; ***p<0.001.

**Table S5.** Pairwise *F*_ST_ (above the diagonal) and Jost’s *D* values (below the diagonal) calculated after excluding loci *Sdu32* and *Sdu46*. Only values highlighted in red were significant when using the full 8-locus dataset. Asterisks (*) denote significance at *p* < 0.05 based on 95% confidence intervals with 10,000 bootstrap replicates.

|  | **BT** | **EE** | **BM** | **BA** | **AN** | **CH** | **CO** | **JF** | **F1BT** | **F1BM** | **F1US** | **F1CL** |
| --- | --- | --- | --- | --- | --- | --- | --- | --- | --- | --- | --- | --- |
| **BT** |  | 0.083* | 0.012 | 0.082* | 0.179* | 0.209* | 0.206* | 0.208* | 0.119* | 0.139* | 0.133* | 0.287* |
| **EE** | 0.084 |  | 0.048* | 0.001 | 0.162* | 0.195* | 0.189* | 0.189* | 0.075* | 0.091* | 0.056* | 0.264* |
| **BM** | 0.004 | 0.044 |  | 0.050* | 0.161* | 0.188* | 0.184* | 0.184* | 0.127* | 0.122* | 0.096* | 0.258* |
| **BA** | 0.109* | 0.001 | 0.035 |  | 0.150* | 0.172* | 0.173* | 0.171* | 0.065* | 0.075* | 0.054* | 0.235* |
| **AN** | 0.584* | 0.492* | 0.606* | 0.548* |  | 0.001 | -0.008 | -0.002 | 0.218* | 0.191* | 0.196* | 0.037* |
| **CH** | 0.569* | 0.545* | 0.609* | 0.544* | 0.001 | A | 0.005 | 0.007 | 0.242* | 0.219* | 0.235* | 0.023* |
| **CO** | 0.613* | 0.540* | 0.631* | 0.591* | -0.013 | 0.000 |  | -0.007 | 0.241* | 0.213* | 0.220* | 0.044* |
| **JF** | 0.546* | 0.471* | 0.545* | 0.509* | 0.000 | 0.008 | -0.011 |  | 0.240* | 0.208* | 0.225* | 0.051* |
| **F1BT** | 0.065* | 0.184* | 0.195* | 0.165* | 0.669* | 0.654* | 0.708* | 0.644* |  | 0.069* | 0.119* | 0.299* |
| **F1BM** | 0.242* | 0.266* | 0.227* | 0.216* | 0.597* | 0.609* | 0.644* | 0.538* | 0.147* |  | 0.113* | 0.280* |
| **F1US** | 0.365* | 0.177* | 0.267* | 0.182* | 0.718* | 0.791* | 0.761* | 0.743* | 0.334* | 0.305* |  | 0.297* |
| **F1CL** | 0.719* | 0.629* | 0.713* | 0.636* | 0.039* | 0.021 | 0.049* | 0.065* | 0.774* | 0.718* | 0.847* |  |

**Table S6.** Analysis of molecular variance (AMOVA) of *Seriola lalandi* in the Pacific.

| Structure tested | Source of variation | Sum of squares | Percent variation | Fixation indices | P value |
| --- | --- | --- | --- | --- | --- |
| Northern Hemisphere  vs Southern Hemisphere | Among groups | 381.931 | 16.92% | *F*_CT_: 0.1692 | 0.0000 |
|  | Among populations within groups | 183.497 | 4.80% | *F*_SC_: 0.0578 | 0.0000 |
|  | Within populations | 3101.328 | 78.27% | *F*_ST_: 0.2173 | 0.0000 |

For the AMOVA analysis, samples were grouped into two geographic clusters: Northern Hemisphere (BT, EE, BM, and BA) and Southern Hemisphere (AN, CH, CO, and JF).

**Figure S1.** Inbreeding coefficients (F_IS_) of 10 groups of *Seriola lalandi* from the northern and southern Pacific. Boxplots represent the median and interquartile ranges of *F*_IS_ values for wild and captive-reared *S. lalandi*, labeled as in Figure 1. Boxplots sharing the same letter differ significantly (*p* < 0.05) according to Dunn’s test with Bonferroni correction.

**Figure S2.** Identification of loci potentially under selection. Loci *Sdu46* and *Sdu32*, initially developed for *Seriola dumerili* (Renshaw et al., 2007), were identified as outliers using a false discovery rate (FDR) threshold of <0.001

**Figure S3.** ΔK values calculated using the Evanno method (Evanno et al., 2005) for the main STRUCTURE analyses: (**a**) all locations from both hemispheres, (**b**) Temperate Northern Pacific locations only, (**c**) locations of wild *Seriola lalandi* in Mexico, and (**d**) Chile. Plots indicate the most likely number of genetic clusters in each group.

**Figure S4.** **(a)** DAPC of 8 wild *Seriola lalandi* sampling locations showing distinct clusters corresponding to the Temperate South America, and Temperate Northern Pacific. Notably, fish from Bahia de Todos Santos (BT) are separate from those in Bahia de Los Angeles (BA) and Ejido Erendira (EE). **(b)** STRUCTURE analysis of 8 wild populations. At *k* = 3, clear genetic differentiation is observed among Mexican populations, while Chilean samples appear genetically homogeneous.

**Figure S5.** Scatterplot of the isolation-by-distance (IBD) analysis among *Seriola lalandi* samples. The Mantel’s test for correlation between *F*_ST_ and log-transformed geographic distance for Temperate Northern Pacific.
